# The authority and politics of epiphanic experience

**DOI:** 10.1007/s42048-026-00250-y

**Published:** 2026-03-30

**Authors:** Matthieu Queloz

**Affiliations:** Institute of Philosophy, University of Bern, Laenggassstrasse 49a, 3012 Bern, Switzerland

**Keywords:** Epiphanies, Phenomenology, Discursive justice, Chappell, Williams, Joyce, Kant, Habermas

## Abstract

In *Epiphanies: An Ethics of Experience, *Sophie Grace Chappell offers a phenomenology of epiphanies—those high points in experience when values most vividly reveal themselves to us. Yet Chappell’s method of using phenomenological descriptions to show that we live by our epiphanies leaves open the question of their *authority*. Why should the epiphanic carry more authority than more sober experiences? The answer, I argue, had better be sensitive to our explanatory understanding of epiphanies. Moreover, it should be sensitive to how the very power of epiphanies threatens to distort our experience of other values. We must beware of what Lichtenberg called *transcendent ventriloquism*, whereby subjective experience is made to sound like something more than what it is—an eternal truth to be enacted at any cost. I then turn to the *politics* of epiphanic experience: how to live together given that we live by different epiphanies. I raise several problems for Chappell’s proposal that the normativity inherent in conversation both imposes transcendental constraints on epiphanic ethics and is determinate enough to prescribe specific political arrangements. These constraints determine either too little or too much; they come too late not to beg crucial questions; they cannot offer an independent check on epiphanic ethics; and they introduce a tension at the heart of the book between the romantic openness to experience, emotion, and enthusiasm and the rationalist aspiration to exclude non-rational influences. This threatens to cast as an illicit intrusion precisely the experiential dimension that epiphanies were to introduce into the conversation.

Once moral philosophy abandons its quest for foundations in human nature or incontrovertible principles of reason, “the only starting point left,” Bernard Williams believed, “is ethical experience itself.” We must begin “from the ways in which we experience our ethical life” (1985, p. 103).

Sophie Grace Chappell’s *Epiphanies: An Ethics of Experience*—a book in constant dialogue with Williams—sets out to show what a philosophy that starts from ethical experience might look like. At the heart of such an ethics of experience, she argues, are *epiphanies*. Why epiphanies? Because epiphanies are “the high points … in the continuum of our subjective experience” (2022, p. 155), “peak-moments … in which value is, or seems to be, particularly clearly revealed to us” (2022, p. 259). If philosophy is to take its bearings from experience, these vivid encounters with value are the first thing it should look to. Paradigmatically, such epiphanies are “sudden and surprising” and “seem to come from outside;” they are “overwhelming” and “take us out of ourselves;” they are “existentially significant,” teaching us “something new” and demanding “a response,” such as love, pity, or creativity; and they are “nourishing,” a kind of “soul food” that sustains the psyche (2022, pp. 8–11, 113–4).

Some will baulk at this conceptual framing, protesting that “epiphany” is not one of their words, as Oscar Wilde might have put it.^1^ But Chappell is clear that while religious revelations are paradigmatic examples of epiphanies, the concept is wider, offering even the non-religious a helpful way of conceptualising “wow-moments” or “aha-moments.” Epiphanies need not be religious in content. They can be experiences of nature, art, or other people. The essential point is that they are peak experiences of value.

In interpreting the notion of epiphany broadly, Chappell aligns herself with James Joyce, for whom epiphanies were fleeting moments of clarity and recognition in which the essence of a situation, character, or relationship radiates out at us.^2^ Joyce undertook to capture such epiphanies in his prose. And Chappell herself draws on literary sources—an impressively rich array of them—to convey the variety and power of the epiphanic.

The book is successful in this, to the point that it makes one wonder why philosophers like Williams, who thought that moral philosophy should start from experience, did not pay more attention to peak experiences. I will address this question in what follows. But the overarching aim of my remarks will be to press Chappell on two main themes: the authority and the politics of epiphanic experience.

A principal thesis of Chappell’s book is that we *live by* our epiphanies. Our ethical sensibilities bear the impress of our epiphanic encounters with value and are disproportionately shaped by these encounters. The claim is meant to be descriptive (73–4). More specifically, it is meant to be a *phenomenological* claim, whose success conditions are that it offers a *sincere*, *accurate*, and *significant* characterisation of what our experience is like—our experience “here and now” (73), primarily, though historical material is adduced to suggest that epiphanies appear across history.

Offering a “phenomenology of epiphanies” (60) is fitting, indeed almost pleonastic in light of the etymology of “epiphany”: as Chappell notes, the Greek *ἐπιφάνεια *originally meant “appearance” (of an object); then “sudden appearance,” as when dawn breaks and light suddenly shines forth (Polybius speaks of the *ἐπιφάνεια *of the day); and then “appearance or revelation of the divine,” which is how Christian theology uses the term. One might add that the German term *Erscheinung *(literally: a *shining forth*) still captures both these immanent and transcendent aspects, and philosophical phenomenology is commonly referred to as *Erscheinungslehre*—roughly, the study of appearances.

It is easy to make too much of such etymological connections. But in suggesting that there is no more fitting object of study for phenomenology than epiphanies, the etymology here motivates a question left open by Chappell, namely to what extent the phenomenological tradition itself ignored epiphanies. According to Chappell, “all philosophy” (56) throughout history failed to acknowledge their significance. Yet her numerous quotations from Scheler, Heidegger, Sartre, Levinas, and Merleau-Ponty (Husserl only makes a brief appearance) suggest that the verdict on the phenomenological tradition will have to be more nuanced, even if they did not discuss peak experiences under the heading of “epiphanies,” but under some different heading.

Precisely because Chappell relies on phenomenological descriptions to substantiate her thesis, however, the book does comparatively little to vindicate the *normative authority *of epiphanies. It may be that, as a matter of fact, we live by our epiphanies. But this does not tell us why we *should *live by our epiphanies—in particular, why they should be regarded as possessing more authority than other ways of relating to value. The book’s subtitle promises an “ethics of experience,” yet what its phenomenological descriptions deliver is perhaps more aptly characterised as an exploration of our most vivid experiences of value. The book is really less about the *ethics* of experience than about the *experience* of ethics.

Admittedly, Chappell does address the authority of epiphanies at one level, namely in granting experience more authority than ethical theory. The theory-builder’s desire to tidy things up, she argues, is like stipulating that *π* = 3. You *can *do that; but it “makes a mess of maths in all sorts of other ways” (66). Similarly, you *can* stipulate that anything which has value has it in virtue of its consequences.^3^ But this makes a mess of ethics in all sorts of other ways: it obfuscates the variety of values that resist such consequentialising; and it encourages one to misconstrue all action as a matter of taking the means to some antecedent end, when much of what we do—playing the violin, smiling at someone, rereading Homer, watching a bee clean its legs—eludes such instrumentalist construals. Philosophers, Chappell observes, “are much too prone to look for systematisations, much too ready to say they *can* (that word again) achieve such systematisations, and as [a] result, much too quick to think that they *can* ignore all the variety and detail that doesn’t fit [into] their systems” (67). To insist on pressing the messy complexity of ethical experience into a tidy theoretical structure “is already to have given up on at least some part of the ‘out-there-ness’ of value” (167).

Epiphanies counteract that tendency. They are striking insights into aspects or dimensions of value that escaped one. To that extent, they tend to *complexify* one’s understanding of the ethical landscape and lead away from a false sense of uniformity.

This suggests a way of sharpening the contrast between “aha-moments” and “wow-moments” that Chappel characterises only by saying that the former is primarily epistemic and the latter primarily evaluative (16, 261). “Aha-moments,” which Chappell also calls “eureka moments,” typically involve a movement from disconnected plurality to unity: as the pieces suddenly fall into place, one sees what holds various phenomena together—one grasps the principle or law that underlies and unifies them.^4^ This kind of understanding involves a form of compression—subsuming overwhelming complexity into a more manageable and efficient representational format.^5^ To that extent, “aha-moments” have a systematising tendency. By contrast, “wow-moments” typically involve a movement in the opposite direction, from simple-minded uniformity to greater variety, density, and richness. This anti-systematic tendency suggests that Chappell’s epiphanies are really more “wow-moments” than “aha-moments.” Echoing Lichtenberg, one might say that epiphanies unmask the majestic simplicity of unifying theories as reflecting nothing more than the rather less majestic simplicity of theorists.

Importantly, the charge against theory-builders is not that they ignore experience altogether; rather, they privilege some parts of experience at the expense of all others. Theory-builders, Chappell quotes Williams as saying, “tend to start from just one aspect of ethical experience, beliefs” (1985, pp. 103–4). This is true of Ross, for instance, who rests his *Foundations of Ethics *on “a large body of beliefs and convictions to the effect that there are certain kinds of acts that ought to be done and certain kinds of things that ought to be brought into existence” (1939, p. 1). Similarly, in a remark that Chappell does not quote, but might well have, Williams concedes that Kant’s moral philosophy “does not overlook everyday human experience;” but he accuses Kant of distorting our view of experience by taking only “part, and a rather local part, of moral experience as its essence,” thereby turning us “away from other kinds of sense we need to make of many other things that are more recalcitrant” (2003, p. 39).

Yet one wonders whether that last remark might not also apply to Chappel’s own account of epiphanies as the heart of ethical experience. For surely, even once it is granted that philosophy should start from experience, there remains a question about how authority should be allocated *within* experience. It is at any rate not obvious why peak experiences should carry *more* authority than calmer, more settled, and less evanescent ways of being in touch with value.

The descriptive claim that people’s ethical sensibilities are *in fact* disproportionately marked by their peak experiences of value is plausible enough—they are, by definition, people’s most vivid, intense, and overwhelming experiences of value. Still, it is one thing to say that we are most impressed by the most impressive, and quite another to say that we are *right* to let these impressions determine the trajectories of our lives. Perhaps we are too impressionable; perhaps we have an infantile tendency to be most impressed by the least important; and perhaps there are good reasons to be suspicious of what is immediately and overwhelmingly awesome.

Chappell recognises that there is a venerable philosophical tradition of being suspicious of affect, ecstasy, fanaticism, and *Schwärmerei*. Her point is that philosophers have tended to *overdo* their privileging of cool detachment over elated engagement. She suggests that we follow Kant in distinguishing *Schwärmerei*, which she translates as fanaticism, from *Enthusiasmus*, participation in the good *with affect*. Affectless detachment may help discern things that full-blooded engagement blinds us to, but “detachment without engagement is empty” (58). As even Kant, that famous *Schwärmer*, acknowledged in his early *Essay on the Maladies of the Head*, “nothing great has ever been accomplished in the world without [enthusiasm]” (AA 2:267). However, in the passage that Chappell cites, which is the *Critique of Judgment*’s much later echo of that pronouncement, Kant actually distanced himself from this view, prefacing it with “it is commonly maintained that” (AA 5:272). The Mongrovius transcription of his 1775–6 anthropology lecture even has Kant rejecting the view altogether with the more characteristic line: “Nowadays enthusiasm is praised so much, but one must intuit principles not with affect, but with cold reason” (AA 25:1287).^6^ There is therefore reason to doubt that Chappell can count on this unlikely ally.

Be that as it may, her own view is that ethics “needs a balance between engagement and detachment” (58). But the question then is how to strike that balance—how much weight to give epiphanic insights against the deliverances of more sober experiences. Chappell leaves that question open.

I suspect that this openness reflects a desire to tiptoe around the issue of epiphanies’ transcendent grounding. Yet how much authority to grant a particular epiphany is an issue that cannot ultimately remain independent from one’s explanatory understanding of that experience. If understood as something bestowed upon oneself by a higher power, the epiphany will appear like a gift purposefully given to one at just that moment. This lends the experience an air of necessity or fatefulness, which in turn imbues the considerations adverted to with authority. As Chappell (384) puts it with Plato, it casts epiphanic insight as knowledge that comes to us *theiai moirai*—by divine allotment. When given a strictly immanent explanation, by contrast, an epiphany is likely to appear deeply contingent: the adventitious product of a momentary susceptibility to this kind of experience happening to be conjoined with the right trigger. Had one not walked by at that precise moment, been in that receptive mood, and seen the scene illuminated in just the right way, the experience would not have been what it was. This casts epiphanic insight as the product of sheer luck.

Chappell makes little of the epistemological difference between these two kinds of reflective understanding, at one point lumping together the idea that epiphanies come to us “by divine grace and favour” with the idea that they do so “by luck” (384). Yet it makes a significant difference whether we are dealing with revelation *theiai moirai* or revelation *by luck alone*, because it affects how much weight we should give to the deliverances of that experience going forward. The exact implications will depend on one’s account of the relevant *theoi* or gods, but whether, say, Hildegard von Bingen’s visions were the product of divine favour or simply of her migraines would certainly have seemed to her like *the *difference.

This is not because immanent explanations imply the *illusoriness* of epiphanies—they had better not, since Chappell compellingly shows that any serious account of ethical experience must accommodate the reality of epiphanic encounters with value. Nor does the fact that Chappell reserves the term “epiphany” for experiences that are revelatory of value prejudge the question of illusoriness, for this “factive” use of the term simply means that the question of illusoriness takes the form of deciding whether what one experienced was indeed a *bona fide* epiphany or just, in Chappell’s terminology, a “*pseud*epiphany” (274).

Rather, the point is that if an epiphany is thought to have a transcendent source, the *context of discovery* can properly support the authority of epiphanic insight in the *context of justification*: the very fact that the evaluative considerations adverted to were discovered through an epiphanic experience imbues them with an authority beyond that which the considerations independently possess. If an epiphany is made sense of in immanent terms, by contrast, authority will have to come entirely from such independent considerations. Moreover, the sheer contingency of the epiphany (Why me? Why now? What if I hadn’t passed by at just that moment?) will cast doubt on its epistemic standing, inviting the worry that such a subjectively variable and unpredictable source of insight is insufficiently sensitive to the truth (the sensitivity condition on knowledge), or at least not safe from error (the safety condition on knowledge).^7^

Another worry about the authority of epiphanies is that their very power to shape the trajectory of our lives might be a problem as much as an answer for ethics. Just because they are overwhelming, epiphanies threaten to *distort *our experience of other values. Their capacity to enthuse and enthral encourages extravagant overreaction (as illustrated by the story of Xerxes becoming so besotted with the beauty of a random tree that he interrupted his campaign, festooned the tree with jewellery and left behind one of his personal guards to watch over it). More ominously, epiphanic experiences can render one insensitive to the *costs* of realising a value. Those who take their moral bearings from one life-altering epiphany too often become single-issue moral crusaders, sacrificing every other value at the altar of their monolithic vision. And the dazzling, overpowering nature of the epiphanic has an inherent tendency to drive off stage the less exciting, but equally important difficulties and costs involved in realising a value in a concrete sociopolitical setting. The very radiance of the epiphanic casts into the shadows the knotty politics of implementation.

Wagner’s *Ring *cycle offers a fitting illustration, because Wagner himself sought to create an operatic experience that was epiphanic, aspiring to orchestrate overwhelming encounters with value that would reshape the trajectory of Germanic culture. This comes out in his tucking the orchestra out of sight to provide a more immersive experience as he designed the *Festspielhaus* in Bayreuth, and of course in the dramatic, grandiose style of the music itself. Indeed, it is the epiphanic character of the music that Williams considered the most problematic aspect of the *Ring*. While other critics focused on its libretto, or on anti-Semitic stereotypes in its character portrayals, Williams believed that this too facilely defanged the problem by externalising it from the music.

What makes the *Ring *feel “peculiarly threatening” (Williams 2006d, p. 87) today is inseparable from the music, Williams contends, because it lies in what the music itself conveys, and in the fact that it so overpoweringly conveys it. The funeral music celebrating Siegfried in *Götterdämmerung*, for example, forcefully underscores the value of a strongman who cuts through the knotty politics of gods and giants. There is value here. But this resplendent, awe-inspiring *mise-en-scène *of a heroic strongman encourages one to forget the *costs* of cutting through the clutter of real politics in this way. The music itself conveys “the suggestion that perhaps there could be a world in which a politics of pure heroic action might succeed, uncluttered by Wotan’s ruses or the need to make bargains with giants … a redemptive, transforming politics which transcended the political” (Williams 2006d, p. 83). As the devastating consequences of this ideal in the century that separates us from Wagner remind us, however, there is real danger in fomenting enthusiasm about the prospect of transcending politics.

Suspicion of the power of the epiphanic to distort one’s experience seems to me an important part of why Williams did not himself focus on peak experiences.^8^ He does cast a spotlight on “such minor revelations of the ethical life as the sense that someone is creepy” (1985, p. 43). And he agrees with Chappell that the aim of moral philosophy “*is* to sharpen perception” (2001a, xv). But he takes “the characteristic fault of perception, at least in moral philosophy,” to be that it “does not go deep enough” (1995b, p. 186). Philosophy should *start* from experience, but it should also retain “the Socratic aim of improving that experience” by liberating the individual “from distortions or misunderstandings involved in his or her own experience” (1995b, p. 218). Nakul Krishna aptly summarises this conception of ethics as “a project directed at the emancipation of our moral experience from the many distortions to which it is vulnerable” (2014, p. 3). On this picture, “we are deceived by forces worth worrying about, such as our own fears and resentments, our misunderstanding of social representations, and the effects of tradition” (Williams 1995b, p. 219). Much of Williams’s work is directed at counteracting these forces, especially by combating their intellectual expressions in ethical theories. He perceived much of the moral philosophy of his day as deploying “notable intelligence … in distorting the facts of human experience” (2014, p. 8) to fit technically sophisticated but fundamentally simple-minded theories, which can make people “feel they have no right to have certain kinds of moral thoughts because they don’t fit in with some very impoverished theoretical picture” (1996b, p. 15)^9^—when in fact, authority flows, if anything, in the reverse direction: it wells up from our ethical experience instead of trickling down from lofty theories.

The other part of the reason why Williams passed over the epiphanic, I suspect, is his wariness of the authority of moral philosophers’ subjective perception. A persistent question for him was, as he put it in an appraisal of Anglophone moral philosophy he wrote in French (the translation is mine): “*By what right* does one, as a philosopher, formulate substantive ethical reflections?” Analytic philosophy, for all its flaws, was acutely aware of a risk that Williams saw well characterised in Jean-François Revel’s *Pourquoi des philosophes? *(1957)—the risk of lapsing into “a psychological or even autobiographical subjectivity that presents itself as the philosophical penetration of some deeper reality at the phenomenological level” (Williams 1980, pp. 878–9). The problem with this, I take it, is not merely that it is tiresomely self-important, but that it arrogates to subjective perception an authority it does not possess. We must beware of what Lichtenberg (1994, I, F 665) called *transcendent ventriloquism*, where some people recount their subjective experiences so solemnly that something said on earth sounds as though it came from heaven.

It is not least out of a concern to avoid this aggrandisement of the subjective that analytic moral philosophy has been animated by “the desire to get away from what is merely ‘ours’” (Williams 2006c, p. 84)—an aspiration made explicit in Hare (1981, p. 17), for example. While this aspiration is taken too far when it aims to wring all that is distinctive out of our cultural situation in a search for timeless foundations, its root concern—to say something that transcends private experience and is recognisable to a wider constituency—is a sound one. That is presumably another part of the reason why Williams tends to proceed by assembling ethical reminders of widely shared and recognisable experiences instead of focusing on more idiosyncratic experiences.^10^

In her conclusion, Chappell in effect acknowledges the danger of transcendent ventriloquism—that some subjective experience might be “idolised,” as she puts it, enforced on others as an eternal truth to be enacted at any cost, threatening to become a source of “fascism” (426). Indeed, a counteracting concern to get away from what is “merely ours” is palpable throughout the book, in Chappell’s emphasis on “the externality, the already-there-ness” (128) of the values we encounter through epiphanies. She cites C. S. Lewis’s observation that attending to your attention to an object precludes attending properly to the object; she invokes Keats’s charge that Wordsworth indulges in the “egotistical sublime,” a mode of writing in which the poet’s subjective state warps the world around him. “One of my central claims,” Chappell stresses, is that “the phenomenology of epiphanies … is not based ‘in our own breasts’, but in the world out there. It is an essential and ineliminable part of this phenomenology that it at least seems to represent how things are” (59–60).

Her main foil for this view is Williams’s internalism about reasons (see especially Chapter 2, § 9, “Against the Internal Reasons Thesis”). As Chappell’s Recognitionalist Manifesto urges: “Values don’t come across to us as any kind of function of our desires or preferences or overall aims, any more than trees that we encounter” (112). The reasons that values generate “are there no matter what is in anyone’s subjective motivational set” (112). Chappell regards philosophical views on which all practical thinking is ultimately self-referential, or “all about me,” as “unattractively self-absorbed” (138). This suggests that the internalism/externalism distinction is the real fault line separating her from Williams.

Yet I do not think that Williams’s internalism is any bar to acknowledging all this. Williams not only *can *make room for phenomenological externality, but *must*, given his other commitments, and *does*, at least in his later work. It would be plainly inconsistent for Williams to hold that the fully articulated first-personal version of practical deliberation must be self-referential, invoking some item in one’s subjective motivational set *S* as *evidence* that one has reason to *ϕ. *This self-absorbed picture, I agree with Chappell, is at odds with the phenomenology of deliberation. Many reasons for action demand to be understood “in a self-forgetting way” (154). To treat the question of whether every human being deserves equal consideration as a function of what is in one’s *S* would be a gross misunderstanding: one’s motivations are neither here nor there in this connection. For Williams’s internalism to dispute this would be for it to ride roughshod over his declaration that philosophy should be answerable to the phenomenology of ethical experience. It would also be hypocritical, going against his own critique of certain forms of utilitarianism, Kantianism, and “virtue theory,”^11^ which he berates for encouraging a degree of preoccupation with one’s own motivations that he denounces as a “reflexive deformation,” a “disproportionate” and “self-indulgent” “misdirection not just of attention,” but “of concern” (1981, pp. 45–49).

Any construal of Williams’s internalism that is true to the rest of his work must accordingly read him as insisting that the agent’s motivations should be *indirectly expressed* in agential deliberation, without necessarily being explicitly *represented *or *referred to* in it. Internalism is not a thesis about self*-representation*, but about self*-expression*. It contends that there should be “a sound deliberative expression of the agent’s *S*” (Williams 2006b, p. 115), but not necessarily under a description that refers to anything in *S*. The agent’s deliberation may still be outward-looking rather than inward-looking, though an external observer—and, in more reflective moments, the agent—may note that *the fact* that these considerations carry weight with the agent *expresses* something of the agent’s *S*. It is *because* an agent has certain motivations that certain facts out there are reasons *for *that agent. But these facts, along with the reasons they generate, may well resist being understood as depending on the agent’s motivations. Just as it is essential to the language game of arithmetic to deny that it depends on human language games (the pupil who says “2 + 3 = 5 *for us*” has misunderstood something), it can be essential to values and concomitant reasons to deny that their force depends on human motivations.

Williams himself stressed this nuance in his later work. “In the situation of decision,” he observes, “the agent’s present motivational state does not primarily act as evidence or support for the conviction that it makes sense for him to act in this way: it is *expressed* in that conviction, just as it is expressed in the action itself” (2002, pp. 236–7). When an agent encounters a fact—the fact that there is a famine, say—and experiences it as being of concern, the agent will have a thought of the form “that is a reason for me to *ϕ.*” But this does *not *mean, Williams emphasises, that the agent “really has, or should really have, the thought ‘that is a reason for me to *ϕ* *in virtue of my S*’.” Rather, “the disposition that forms part of his *S* just is the disposition to have thoughts of the form ‘that is a reason for me to *ϕ*’, and to act on them” (2001b, p. 93). Some reasons are correctly understood only if they are understood to be *unhintergehbar, *as he puts it—“there is nothing more basic in terms of which to justify [them]” (2006a, p. 195), and *a fortiori* nothing in our *S*. These reasons are “simply there” (2006a, p. 195), even when we know, at a reflective level, that they are only simply there *for us*, while past societies were insensitive to them. But precisely what it *is* for a reason to be, for us, simply there, on his account (2006a, p. 195), is not for us to have the thought: “For us, it is simply there.” It is for us to have the thought: “It is simply there.”

Here, Williams sounds exactly like Chappell, who opens her Manifesto with the declaration that values are “just there” (112). Williams can also agree with Chappell that “our human phenomenology presents various aspects or dimensions of the world to us, not neutrally or dispassionately, but as *of concern to us*” (166). The point of internalism is merely that this fact itself reflects something about our motivations, even when these do not figure in our deliberation, because they play a role in *making it the case *that these aspects of the world are of concern *to us*. As I have argued elsewhere, internalism is not a normative claim enjoining us to egocentrically consider how to further our own motivations. It is a metaethical claim, meant to help us make *explanatory* sense of the fact that different considerations make normative sense to different people.^12^ This is perfectly compatible with an externalist phenomenology of deliberation (indeed, as Williams’s later genealogical inflection of internalism shows, internalism can even explain why some values, like the value of truth, *need *to be experienced in externalist terms).

I turn, finally, from the authority to the politics of epiphanic experience. Towards the end of the book, Chappell asks “how we are to live together, given that we live by different epiphanies” (412). Her answer appeals to a transcendental argument in a Habermasian vein: the conditions and presuppositions of conversation themselves yield normative standards—the “norms of conversational justice” or “Ten Socratic Commandments”—because “to engage in rational exchange with others at all is to incur a rational commitment to eschew the techniques of *non-rational* exchange” (411). She takes the ramifications of this commitment to show that “the right political ideal for modern liberal humanists” (411) is to aim at “any of the multiplicity of possible conversational republics”, where a *conversational republic* is a social arrangement that accords with the norms of conversational justice—“a social and political space within which all, as equals, are free to try and persuade each other of the truth of their own epiphanies by whatever legitimate means of persuasion they like” (414).

On this view, there is a normativity inherent in conversation that imposes transcendental constraints on *which* epiphanies can be considered and *how*. Any epiphany mandating something that goes against the norms of conversation will be “ruled out of consideration by conversational justice,” “because the whole idea of our having a *conversation* about epiphanies is that the option of violently coercing anyone is rejected before we start” (413). Similarly, any epiphany that “systematically marginalises” certain groups offends against conversational justice, “which requires you to speak and (equally crucially) listen in the conversation only in ways that treat as equal conversational partners everyone involved” (413). Only those epiphanies count that are suited to being the objects of *rational* exchange, exchange that is free from distortion by *non-rational* forces.

How much do these transcendental constraints determine? The question is muddied by the fact that Chappell seems to slide from fairly weak premises—Socratic injunctions to eschew rhetorical tricks from the Sophists’ toolbox, and Gricean norms of brevity and relevance—to remarkably substantial political conclusions, such as a commitment to equal treatment and recognition, and to mutual non-domination between equal citizens in a Pettit-style republic. This feels like Chappell is trying to squeeze more out of the Ten Socratic Commandments than they carry in them.

But the fundamental problem with this strategy is that the transcendental constraints are in danger of determining either too little or too much. They determine *too little* if they merely exclude epiphanies that are incompatible with having a conversation. This formal idea still leaves open almost every political question about how we are to live together, just as shutting the anarchists out of the political conversation still does nothing to tell remaining participants *how* to organize and deploy the state power they accept.

On the other hand, if the transcendental constraints are beefed up sufficiently to deliver substantive political commitments, as they do in discourse ethics, they risk determining *too much* for Chappell’s purposes: in order to yield something as strong as *transcendental* constraints, the norms of conversation must be independent of the contents of experience, which means that the various revelations of epiphanic experience will be subordinated to them. Once one admits into the picture constraints that are simultaneously transcendental and determinate enough to prescribe something as specific as a political arrangement in which people are free and equal and no group is marginalised or dominated, those constraints are apt to take over the show. And then we are back to pressing the complexities of experience into a tidy rational system.

Chappell’s rationale for turning to these transcendental constraints was to counter the worry that epiphanies, particularly of the religious kind, can breed fanaticism and violence. But appealing to the preconditions of conversation at this point seems to presuppose what it is meant to justify: that the people whose epiphanic experiences issue in violent frenzy *are* committed to realising their values through demure conversation. However weak or strong the norms of conversational justice end up being, they must come too late not to beg that question.

Another problem is that, as Williams urged against Habermas, distinguishing between rational and non-rational forces cannot ultimately remain a matter of enforcing a technical distinction, like clearing a signal from noise, but is itself an ethical and political matter we continually need to renegotiate on the basis of our values.^13^ Even the clearest instantiations of rational conversation still take place within relations of power, and can never entirely exclude the effects of supposedly non-rational factors such as mood, affect, felicity of expression, oratory skill, charisma, or social status.

The point is particularly clear with conversations about epiphanies. What is the conversation supposed to be animated by, if not the non-rational impression made upon one by the experience? An epiphany is not an argument. We do better to see even the most rational conversation about epiphanies as something that is partly motivated, sustained, and constituted by non-rational forces, and to regard the distinction between *constructive* and *distorting* forces as a distinction that needs to be drawn *within* a spectrum that always combines reason and power to varying degrees, and never presents us with unadulterated reason. We must then negotiate which forms of non-rational influence to accept—and where: in the sphere of education, for example, we happily accept forms of coercion, emotional manipulation, and motivation by external incentives that we resist in *bona fide *democratic politics. However, as recent political history reminds us, where we draw these lines is itself something that we continually renegotiate, and that is highly sensitive to who “we” is.

For Chappell’s account, this would mean that the constraints of conversational justice, once made fully determinate, will be less *transcendental* than *social*: they can offer no completely independent check on an ethics informed by experience, since that experience will itself have to inform how we draw the distinction between legitimate and illegitimate means of persuasion.

Taken together, these observations suggest that there is a tension at the heart of the book. In putting epiphanies at the heart of ethics, the book displays a romantic openness to experience and the emotions and enthusiasm it unleashes. But in treating the Ten Socratic Commandments as transcendental constraints on epiphanic insights, and in ruling out advocacy for those insights that involves non-rational factors such as “manipulating your hearers’ emotions” (413), the book displays a rationalist streak which ends up subordinating epiphanic ethics to discourse ethics. There is a tension between the romanticism and the rationalism in this line of argument. The norms of conversational justice threaten to cast as an illicit intrusion precisely the experiential dimension of the ethical that epiphanies were supposed to introduce into the conversation.

Perhaps that is a tension we simply have to live with.^14^ But perhaps a more promising way of moving from the ethics to the politics of epiphanic experience would be to acknowledge, in more concrete terms, the specific political difficulties involved in making rules that are responsive to particular types of epiphanic experience. Williams once remarked that what makes much moral philosophy feel “frivolous” in how it approaches serious subjects such as abortion is its “refusal to engage with the only two things that matter: the politics of trying to make rules for such situations, and the experience of people engaged in them” (1995b, p. 221n10). Chappell’s magisterial book performs an extremely valuable service in reminding us of how eye-opening, catalytic, and transformative epiphanic experiences can be, and how central a place that fact deserves to hold in moral philosophy. But that is exactly half the battle. The other half consists in moving from these life-changing experiences of the ethical to the politics of experience, and engaging realistically with what must appear, by comparison, rather anti-epiphanic: the slow and cumbersome grind of finding compromises and formulating policies and regulations for the social world in which we live our epiphany-enthused lives.^15^

## Footnotes

1 Challenged in court to deny that a story was blasphemous, Wilde retorted that “blasphemous” was not a word of his (Hyde 1973, p. 107).

2 Joyce (1944, pp. 216–8).

3 Though see Williams (1973, p. 82) on the regress problem this generates.

4 See, e.g., Kitcher (1981).

5 See Wilkenfeld (2019).

6 On how Kant came to distance himself from Shaftesbury and Rousseau on this point, see Clewis (2018).

7 On the sensitivity condition, see Nozick (1981, ch. 3). On the less demanding and more popular safety condition, see Sosa (1999), Williamson (2000), and Pritchard (2007).

8 Another factor may be his deep aversion to sentimentality and kitsch—the avoidance of which can be a challenge in depicting epiphanies; see Krishnan and Queloz (2023).

9 See Williams (1995b, p. 218, 2021, p. 275).

10 On the Wittgensteinian echoes of Williams’s method, see Queloz and Krishnan (2025).

11 I put “virtue theory” in scare quotes because Williams (1995a, p. 551, 1996a, p. 27, 1998) emphasises that a virtuous person needs *virtuous dispositions*, not a *theory* about them, and to possess these dispositions is not, typically, to be disposed to think about one’s own or indeed anyone’s virtuous motives, but rather for “ranges of fact” to become “ethical considerations” for one *because* one has those dispositions (1985, p. 11). For a complementary critique of the tendency to treat virtue ethics as the “third kid on the block,” on a par with deontology and consequentialism, see Halbig (2020).

12 See [Anonymised]. As I argue there, this does not mean that internalism makes no difference to practice—it can inform deliberation indirectly, and forms the philosophical underpinning of Williams’s liberalism.

13 See Williams (1995c, p. 148, 2002, p. 226).

14 I am indebted to an anonymous reviewer for suggesting this possibility.

15 I am grateful to Christoph Halbig as well as to two anonymous reviewers for helpful comments and suggestions on an earlier draft.
